# LDSNet: A Lightweight Detail-Sensitive Network for Small Object Detection in Low-Altitude UAV Scenarios

**DOI:** 10.3390/jimaging12050209

**Published:** 2026-05-14

**Authors:** Tong Tan, Xianrong Peng, Jianlin Zhang, Haorui Zuo, Yao Zhang, Yunhao Wu, Hui Li

**Affiliations:** 1 State Key Laboratory of Optical Field Manipulation Science and Technology, Institute of Optics and Electronics, Chinese Academy of Sciences, Chengdu 610209, China; 2 University of Chinese Academy of Sciences, Beijing 100049, China

**Keywords:** Unmanned Aerial Vehicle (UAV), small object detection, lightweight network, real-time inference

## Abstract

Object detection in Unmanned Aerial Vehicle (UAV) imagery faces significant challenges due to the unique aerial perspective. A major bottleneck is the weak feature representation of small objects, which limits both detection accuracy and computational efficiency. To address this issue, we propose a Lightweight Detail-Sensitive Network (LDSNet). Specifically, LDSNet consists of three key components: (1) Lightweight Detail-Sensitive Downsampling (LDSDown), which combines anti-aliasing smoothing with dual-path feature extraction to preserve the spatial details of small objects during downsampling; (2) Shared Recursive Dilated Convolution (SRDC), which uses weight-shared multi-rate dilated convolutions to capture multi-scale context and enlarge the receptive field without introducing extra parameters; and (3) Deeply Decoupled Grouped Head (DGHead), which employs high-ratio grouped convolutions to significantly reduce the computational cost of processing high-resolution inputs. Extensive experiments on the VisDrone2019 and HIT-UAV datasets demonstrate that LDSNet achieves an excellent trade-off between accuracy and efficiency. Compared to the YOLOv11n baseline, LDSNet reduces parameters by 84.6% (from 2.6 M to 0.4 M) and FLOPs by 29.2% (from 6.5 G to 4.6 G), while improving mAP_50_ by 2.2% on VisDrone2019 and achieving 94.5% on HIT-UAV.

## 1. Introduction

The rapid development of Unmanned Aerial Vehicle (UAV) platforms and the decreasing cost of hardware have established low-altitude remote sensing as a core modality for geospatial data acquisition [1,2]. This technology plays an indispensable role in domains such as smart city traffic surveillance [3], maritime search and rescue [4], precision agriculture, and military reconnaissance [5]. Unlike stationary ground sensors or satellite imagery, UAVs offer superior mobility and a panoramic perspective [6]. While general-purpose object detectors, typified by the YOLO series [7,8], have achieved superlative performance on natural scenarios datasets [9], their direct deployment in UAV-captured aerial scenarios remains hindered by substantial scale disparities [10].

Under defined low-altitude ranges (typically 30–150 m) and specific camera configurations (e.g., wide-angle lenses with high-resolution sensors), the small object category exhibits unique physical and visual characteristics, including extremely limited pixel footprints, a complete absence of internal textural details, and a high dependency on regional environmental logic for disambiguation. The core scientific challenge in UAV-based detection lies in the insufficient feature representation capability for these infinitesimal objects, which manifests primarily in two sub-problems:

(1) Severe information loss during the downsampling of tiny objects: Owing to the elevated vantage point, objects exhibit drastically reduced pixel footprints, often occupying fewer than 16×16 pixels. This “pixel poverty” makes the geometric and textural structures of tiny objects extremely fragile [8,11].
(2) Insufficient global feature representation constrained by local receptive fields: Because tiny objects inherently lack distinct internal visual features and often suffer from dense distribution and mutual occlusion, their accurate identification relies heavily on broad environmental co [12,13].

Existing methodologies struggle to reconcile feature representation efficacy with computational efficiency when addressing these two sub-problems [5]. To combat the fragility of tiny objects, traditional downsampling operations are commonly used; however, they often cause coarse spatial aliasing and lead to the irreversible “semantic annihilation” of small objects in deep layers [14,15]. Alternative downsampling strategies have also been proposed: SPD-Conv employs space-to-depth combined with non-strided convolution [16], and Haar wavelet downsampling applies wavelet decomposition while retaining the low-frequency component [17]. Furthermore, to address the need for global context and overcome restricted receptive fields, various context-aggregation mechanisms have been developed. Standard parallel architectures (such as ASPP) or complex attention mechanisms are frequently employed to expand the receptive field. Recent advancements include the AIFI module, which applies multi-head self-attention to high-level features for intra-scale feature interaction and long-range dependency modeling [18]. Similarly, modules like SPPF-LSKA integrate a spatial pyramid pooling structure with large separable kernel attention to capture broader contextual cues [19]. As a direct architectural countermeasure to preserve fine-grained spatial details and improve small-object recall, recent detection networks frequently introduce shallower, high-resolution feature layers (e.g., the P2layer) [20,21]. Unfortunately, while this explicit retention of high-resolution semantics effectively mitigates the fragility of tiny objects, processing these massively scaled feature maps imposes a severe computational burden.

To address these limitations, this paper propose the Lightweight Detail-Sensitive Network (LDSNet). LDSNet is designed as a unified architectural framework specifically to tackle the core challenge: the insufficient feature representation capability for tiny objects. This central problem is further decomposed into two interrelated sub-problems: the loss of fine-grained spatial information during downsampling, and the difficulty of capturing broad regional context at high resolutions. We perform a macro-architectural reconfiguration-integrating a high-resolution P2 layer while omitting the deep P5 layer to redirect the parameter budget toward infinitesimal targets. Within this re-engineered hierarchy, three synergistic modules are introduced to execute this logic:

Firstly, to address Sub-problem 1, this paper designs the Lightweight Detail-Sensitive Downsampling (LDSDown) module. LDSDown synergizes an anti-aliasing preprocessing mechanism acting as a spatial low-pass filter with a dual-path heterogeneous extraction strategy. This approach explicitly safeguards the spatial integrity of tiny objects against decimation artefacts during downsampling while significantly minimising the computational load. Secondly, to address Sub-problem 2, this paper proposes the Shared Recursive Dilated Convolution (SRDC) module. By replacing traditional deterministic pooling with a cascaded sequence of multi-rate dilated convolutions sharing a unified weight tensor, SRDC dynamically expands the effective receptive field without degrading spatial resolution. This lightweight mechanism empowers the network to extract multi-scale regional semantics, seamlessly coupling minute objects with their global context at zero additional parameter cost. Lastly, to alleviate the computational surge induced by the high-resolution P2 stream, the Deeply Decoupled Grouped Head (DGHead) operates as an efficiency engine that linearizes complexity through high-ratio grouped convolutions. This cohesive pipeline ensures that the enriched detail and context can be effectively translated into precise predictions while strictly adhering to real-time constraints.

This philosophy reframes the accuracy-efficiency trade-off as a strategic resource reallocation. By pruning the deep P5 layer which offers little benefit for tiny targets, we recover a substantial parameter budget and reinvest it into the high-resolution P2 stream where fine details are most visible. Within this framework, each module plays a specific role in maintaining this balance: LDSDown protects spatial precision with a minimal footprint, SRDC adds global context at zero parameter cost, and DGHead streamlines high-resolution processing for real-time speed. This synergy ensures that every unit of computation is maximized, allowing LDSNet to remain exceptionally lean while sharpening its sensitivity to infinitesimal targets.

The primary contributions of this work are summarised as follows:

- To address the severe information loss of tiny objects during downsampling, this paper propose a lightweight architecture, LDSNet, featuring the Lightweight Detail-Sensitive Downsampling (LDSDown) module. By leveraging anti-aliasing preprocessing and a dual-path heterogeneous extraction strategy, it successfully preserves the fragile geometric structures of infinitesimal objects while drastically minimizing parameter overhead.
- To overcome the constraints of local receptive fields and the computational bottlenecks of high-resolution feature maps, this paper design the Shared Recursive Dilated Convolution (SRDC) and Deeply Decoupled Grouped Head (DGHead) modules. SRDC dynamically expands the receptive field via weight-shared recursive convolutions to supplement global semantics at zero parameter cost, while DGHead utilizes grouped convolutions to dramatically reduce FLOPs, achieving an optimal trade-off between detection fidelity and inference efficiency.
- Extensive experiments on broad UAV datasets (VisDrone2019 and HIT-UAV) demonstrate that LDSNet achieves a highly favorable balance between accuracy and efficiency. Compared to the YOLOv11n baseline, LDSNet realizes an 84.6% reduction in parameters (from 2.6 M to 0.4 M) and a 29.2% reduction in FLOPs (from 6.5 G to 4.6 G), while concurrently improving mAP_50_ by 2.2% on VisDrone2019 and reaching 94.5% on HIT-UAV.

## 2. Related Works

### 2.1. Evolution of Paradigms in Remote Sensing Object Detection

The methodology for identifying objects in remote sensing has evolved from manual feature engineering toward deep learning-based data-driven paradigms [22]. Early methodologies employed manual descriptors such as HOG and SIFT [23]. While adequate in constrained environments, these features lacked the representational capacity to handle the intricate backgrounds, variable viewpoints, and scale fluctuations typical of remote sensing scenarios [24]. Currently, deep learning approaches are dominated by two primary lineages: Convolutional Neural Networks (CNNs) and Transformers [25,26].

(1) CNNs: Two-stage frameworks (e.g., Faster R-CNN) prioritise precision via RPNs but suffer from heavy computational latency [27], making one-stage detectors like YOLO the preferred choice for time-critical UAV missions [28,29].
(2) Transformers: the DETR-based Transformer paradigm offers end-to-end detection by removing NMS, yet the vanilla DETR is plagued by slow convergence and insensitivity to small-scale features, limiting its airborne utility [30,31].

### 2.2. Small Object Detection Strategies in UAV Perspectives

In UAV imagery, object often occupy fewer than 32×32 pixels, making them susceptible to feature loss during downsampling. Contemporary research addresses this through three main strategies:

(1) Multi-scale Feature Fusion and High-Resolution Retention: To prevent information loss in deep layers, CNN-based methods employ architectures like FPN, PANet, or BiFPN to aggregate shallow spatial details with deep semantics [32]. For example, FFCA-YOLO utilises channel re-weighting to enhance fusion [33], while LMSFA-YOLO and YOLO-CAM integrate high-resolution detection heads (e.g., P2) specifically for minute objects [34,35]. Particularly in high-resolution UAV images, the disparity between large-scale backgrounds and infinitesimal targets is further exacerbated. To tackle this, slicing-based strategies like SAHI [30] facilitate multi-scale inference by partitioning large frames into overlapping patches. Furthermore, asymmetric multi-scale fusion and adaptive feature sampling mechanisms, such as those in QueryDet [36] and MBD [6], focus computational efforts on high-resolution regions containing small objects, effectively reconciling the demand for fine-grained localization with wide-area perception. Similarly, SSABNet employs spatial-semantic aggregation to ensure high-fidelity feature restoration for small UAV objects [37]. These approaches often remove redundant deep heads to reduce parameter volume while maintaining detail.
(2) Context-Awareness and Receptive Field Optimisation: Given the limited information in small object, exploiting context is critical. Techniques such as the Multi-Kernel Perception (MKP) unit in FBRT-YOLO use serial convolutions of varying sizes to capture multi-scale context [38]. Similarly, DAU-YOLO employs Receptive Field Attention (RFA) [39], and other works utilise large-kernel convolutions to distinguish object from background noise effectively [40].
(3) Transformer Query Optimisation and Relational Reasoning: In DETR-based models, standard object queries often fail to localise small object. Methods like SMCA-DETR and Anchor DETR address this by introducing spatial priors or explicit anchor associations, thereby significantly accelerating convergence and improving recall for diminutive objects [41,42]. Beyond basic queries, recent Transformer advancements leverage dynamic token interactions and relational reasoning for complex aerial scenarios. For instance, HGINet utilises hierarchical graph interactions and token clustering to capture the fine-grained semantics of camouflaged objects [43]. Furthermore, to combat severe occlusions caused by extreme weather, visual relationship reasoning frameworks like CTRP [44] and DGRL [45] exploit global relational contexts, offering robust solutions for adverse environments.

### 2.3. Model Lightweighting

Constrained by finite battery endurance and computing power, detection models for UAV must emphasise parametric efficiency. Lightweighting efforts generally focus on architectural design and post-processing.

(1) Lightweight Backbones and Efficient Operators: Early works compressed models by adopting backbones like MobileNet [46]. Recent research focuses on specialised operators, such as Depthwise Separable Convolutions (DWConv) in MobileYOLO or Partial Convolutions (PConv), to reduce redundancy [47]. In the Transformer domain, architectures like MobileViT and EfficientFormer aim to reconcile the global perception of ViTs with the low latency required by edge devices [48,49].
(2) Model Compression and Inference Optimisation: Techniques such as knowledge distillation [50], network pruning [51], and quantisation (e.g., FP32 to INT8) reduce model size for edge deployment without altering the architecture [52]. Furthermore, optimising post-processing (e.g., replacing standard NMS with Soft-NMS-SIoU) can enhance precision for dense object groups while maintaining algorithmic efficiency [53].

## 3. Proposed Methods

### 3.1. Overall Architecture

**Figure 1:**
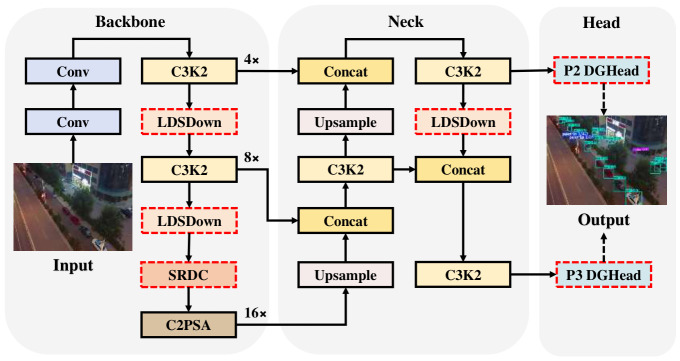
The overall architectural framework of the proposed LDSNet. The red dashed boxes highlight the core modular innovations, including LDSDown, SRDC, and DGHead.

To address the challenges posed by infinitesimal object scales, dense distributions, and cluttered backgrounds in Unmanned Aerial Vehicle (UAV) remote sensing imagery, this paper proposes the Lightweight Detail-Sensitive Network (LDSNet), a lightweight and efficient detection network. Its overarching architecture is illustrated in Figure 1. Rather than a mere assembly of standalone components, LDSNet is designed as a unified architectural framework. Following the conventional “Backbone–Neck–Head” design paradigm [54], LDSNet is built on the YOLOv11 framework but undergoes a profound structural reconfiguration tailored to the physical attributes of small object.

As shown in Figure 1, the model architecture is partitioned into the Backbone for feature extraction, the Neck for multi-scale feature fusion, and the Head for object prediction. The data flow originates from the input image and culminates at the final detection output. The network incorporates standard convolutional (Conv) layers, C3K2 (Cross Stage Partial Bottleneck) stages for feature representation, and a C2PSA (CSP with Programmable Spatial Attention) module to capture focused spatial context. Within the Neck, Upsample and Concatenation (Concat) operations are utilized to achieve bidirectional feature integration.

Firstly, this paper performed a structural reconfiguration of the backbone architecture. Conventional YOLO networks typically extend downsampling to the P5 layer (with 32× downsampling) [35,55]. However, because minute objects in UAV perspectives occupy an exceedingly low pixel count, often below 32×32, excessive subsampling frequently precipitates the “semantic annihilation” of critical features. Consequently, LDSNet restricts its downsampling depth to the P4 layer (with 16× downsampling) to preserve a higher-resolution feature flow. To improve feature extraction quality, the Lightweight Detail-Sensitive Downsampling (LDSDown) module is integrated. It combines anti-aliasing smoothing with a dual-path strategy to safeguard spatial integrity while suppressing sampling artefacts. Furthermore, the Shared Recursive Dilated Convolution (SRDC) module is embedded at the backbone’s terminus. By sharing weights across different dilation rates (d∈{1,3,5}), SRDC extracts features at multiple scales and expands the receptive field. This effectively compensates for the removal of the P5 layer (with 32× downsampling) without adding any extra parameter overhead. This ensures that the backbone provides the Neck with features that are both spatially sharp and semantically rich.

Secondly, the feature fusion network (Neck) was optimised to better accommodate the characteristics of small object. Utilising a bidirectional fusion architecture inspired by PANet, the fusion logic is shifted “upward” through cascaded upsampling to integrate a high-resolution P2 feature layer (with 4× downsampling). This provides an abundance of textural cues essential for the precise localisation of infinitesimal object. Within the bottom-up augmentation pathway, the LDSDown module is employed to facilitate anti-aliasing and information compensation during the aggregation of multi-resolution feature maps, thereby minimising spatial distortion and redundancy during the fusion process. This module serves as the bridge that maintains detail consistency as features transition from local textures to regional context.

Thirdly, the detection heads were reorganised and streamlined to strike an optimal balance between architectural economy and precision. LDSNet discards the redundant P4 and P5 heads and focuses instead on dual-scale branches at the P2 and P3 levels. This strategic reconfiguration significantly improves recall for densely distributed small objects. To manage the substantial computational load incurred by the high-resolution P2 layer, this paper developed the Deeply Decoupled Grouped Head (DGHead). By reconstructing the Stem layer with high-group-rate convolutions, DGHead effectively curtails redundant cross-channel interactions and alleviates computational pressure, ensuring the model maintains real-time inference efficiency. Ultimately, LDSNet markedly improves perceptual sensitivity to minute object while simultaneously achieving an exceptionally lean parameter footprint. By thoroughly decoupling classification and localization tasks, DGHead ensures that the detail-sensitive features preserved by LDSDown and the regional context provided by SRDC are efficiently utilized for robust object detection.

### 3.2. Lightweight Detail-Sensitive Downsampling (LDSDown)

**Figure 2:**
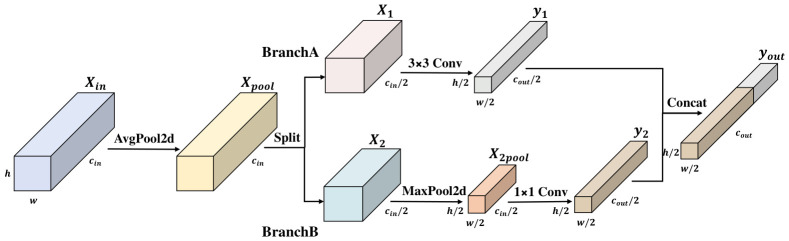
Structure of LDSDown.

UAV-based remote sensing requires detection frameworks that can effectively harmonise stringent precision requirements with the need for real-time responsiveness [5]. On the one hand, benchmark datasets such as VisDrone2019 are characterised by minuscule object scales and intricate background textures, necessitating the preservation of high-resolution, fine-grained spatial information during downsampling [30]. Conventional downsampling techniques, such as standard strided convolutions or max pooling, often suffer from coarse spatial sampling, which induces aliasing and compromises the geometric structural integrity of object. On the other hand, although the YOLOv11 architecture achieves superlative feature extraction performance, its reliance on standard strided convolutional modules incurs substantial parameter overhead and computational redundancy [55]. To address these limitations, this study introduces the Lightweight Detail-Sensitive Downsampling (LDSDown) module, as illustrated in Figure 2. By synergising anti-aliasing preprocessing with a dual-path heterogeneous extraction strategy, LDSDown achieves a superior equilibrium between detail retention and computational efficiency.

The operational workflow of the LDSDown module is systematically organized into four sequential stages to ensure information integrity during spatial reduction:

Stage 1: Anti-aliasing Preprocessing. To counteract the high-frequency artefacts and feature distortion inherent in direct sparse sampling (i.e., immediate strided decimation), LDSDown initiates with a deterministic anti-aliasing phase. Specifically, the input feature map $X_{\mathrm{in}}\in R^{c_{\mathrm{in}}\times h\times w}$ is processed via a non-learnable 2×2 average-pooling operator. By strategically employing a stride of s=1, the spatial resolution (h×w) is strictly preserved. Mathematically, this acts as a spatial low-pass filter that smooths out high-frequency noise before spatial decimation, establishing a stabilised intermediate tensor $X_{\mathrm{pool}}$ without adding learnable parameters.
Stage 2: Channel Splitting. To mitigate informational bottlenecks and minimise the computational workload (FLOPs), a channel-partitioning strategy is implemented. The pre-processed feature map $X_{\mathrm{pool}}$ is uniformly split into dual sub-tensors, $X_{1}$ and $X_{2}$, effectively halving the input dimensionality for subsequent convolutional operations:
  (1) $$X_{1},X_{2}=\mathrm{Split}\left(X_{pool},dim=1\right),\;X_{1},X_{2}\in R^{\frac{c_{in}}{2}\times h\times w}$$
Stage 3: Dual-path Differentiated Feature Extraction. This stage represents the core innovation of LDSDown, utilizing two heterogeneous pathways to extract distinct feature attributes:
  - Local Texture Enhancement Path (Branch A): This branch employs a 3×3 convolutional operation with a stride of s=2 to encapsulate local spatial details. The output $y_{1,k,i,j}$ is computed via the discrete cross-correlation summation:
    (2) $$y_{1,k,i,j}=\sum_{c=0}^{\frac{c_{\mathrm{in}}}{2}-1}\sum_{m=0}^{2}\sum_{n=0}^{2}W_{k,c,m,n}\cdot x_{1}\left(c,2i+m-1,2j+n-1\right)+b_{k}$$
  - Saliency Feature Compensation Path (Branch B): This pathway integrates 3×3 strided max-pooling (s=2) with a 1×1 pointwise convolution to isolate prominent spatial responses. The final output $y_{2,k,i,j}$ is formulated as:
    (3) $$y_{2,k,i,j}=\sum_{c=0}^{\frac{c_{\mathrm{in}}}{2}-1}W_{k,c}^{\prime }\cdot \left[\max_{m,n\in \{0,1,2\}}x_{2}\left(c,2i+m-1,2j+n-1\right)\right]+b_{k}^{\prime }$$
  This dual-path configuration is predicated on the principle of information complementarity under strict sampling constraints. By bifurcating the extraction process, Branch A functions as a learnable adaptive filter to retain complex textural manifolds, while Branch B acts as a non-linear saliency detector. Since infinitesimal UAV targets often manifest as isolated intensity peaks, the max-pooling pathway ensures that these critical impulses are not dissipated by the smoothing effect of the preceding anti-aliasing phase, thereby sustaining a high signal-to-clutter ratio.
Stage 4: Feature Fusion. The sub-features from both paths are aggregated via channel-wise concatenation to form the final downsampled output $y_{out}$. This process restores the channel depth while coupling rich textural details from Branch A with the salient structural characteristics from Branch B.

To quantify efficiency, the complexity of LDSDown is benchmarked against the original 3×3 strided convolution in YOLOv11. Let the input and output channel counts be $c_{in}$ and $c_{out}$, respectively, and the feature map dimensions be h×w.

The parameter count ($P_{std}$) and computational load ($F_{std}$) for the standard convolution are formulated as:

(4) $$P_{std}=9c_{in}c_{out},\;F_{std}=2.25hwc_{in}c_{out}$$

In LDSDown, the parameter count ($P_{LDS}$) and computational complexity ($F_{LDS}$) are derived as follows:

(5) $$P_{LDS}=\underset{BranchA}{\underset{︸}{\left(3^{2}\cdot \frac{c_{in}}{2}\cdot \frac{c_{out}}{2}\right)}}+\underset{BranchB}{\underset{︸}{\left(1^{2}\cdot \frac{c_{in}}{2}\cdot \frac{c_{out}}{2}\right)}}=2.5c_{in}c_{out}$$

(6) $$F_{LDS}=\underset{BranchA}{\underset{︸}{\left(\frac{h}{2}\cdot \frac{w}{2}\cdot 9\cdot \frac{c_{in}}{2}\cdot \frac{c_{out}}{2}\right)}}+\underset{BranchB}{\underset{︸}{\left(\frac{h}{2}\cdot \frac{w}{2}\cdot 1\cdot \frac{c_{in}}{2}\cdot \frac{c_{out}}{2}\right)}}=0.625hwc_{in}c_{out}$$

Theoretical analysis indicates that LDSDown achieves a significant 3.6-fold reduction in both parameters and FLOPs ($P_{std}/P_{LDS}=F_{std}/F_{LDS}=3.6$). Consequently, by drastically reducing computational burden while enhancing feature awareness, LDSDown effectively enhances the model’s capacity for detailed object detection.

### 3.3. Shared Recursive Dilated Convolution (SRDC)

For object detection in aerial imagery, models face an inherent tension between preserving spatial resolution and expanding the effective receptive field. Since object in datasets like VisDrone2019 possess exceedingly sparse pixel footprints, retaining high-resolution, fine-grained information is imperative [56]. Conventional Spatial Pyramid Pooling-Fast (SPPF) modules suffer from deterministic information loss due to non-learnable pooling, frequently discarding the essential textural details of tiny object [55]. Conversely, removing deep downsampling layers (e.g., the P5 layer) safeguards resolution but restricts the network’s capacity for regional semantic association. While dilated convolutions can broaden the receptive field without resolution degradation, standard parallel architectures (such as ASPP) incur substantial parameter and computational overhead [57].

**Figure 3:**
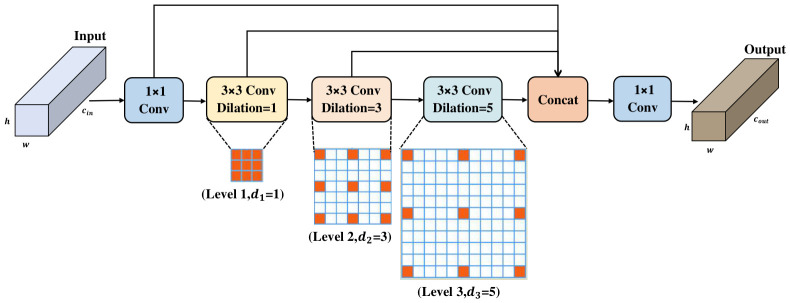
Structure of SRDC. The orange squares in the grids denote the active sampling locations of the 3×3 convolutional kernels, illustrating the progressively expanding receptive fields corresponding to different dilation rates ($d_{1}=1$, $d_{2}=3$, and $d_{3}=5$).

To circumvent these limitations, this study proposes the Shared Recursive Dilated Convolution (SRDC) module (Figure 3). SRDC replaces traditional pooling with a tiered convolutional architecture to precisely capture fine-grained details and regional semantics. By leveraging recursive cascading and a weight-sharing mechanism, SRDC achieves dynamic receptive field expansion and multi-scale feature integration with negligible additional overhead.

The SRDC module comprises four functional segments: channel compression, a recursive dilated convolutional sequence, feature aggregation, and output reconstruction.

Channel Compression: Initially, the input feature map $X\in R^{c_{in}\times h\times w}$ is processed via a 1×1 convolution to aggregate cross-channel information and reduce dimensionality. This yields a compact base feature representation, $F_{base}\in R^{c^{\prime }\times h\times w}$ (where $c^{\prime }=c_{in}/2$), which serves to minimise the computational workload for the subsequent recursive operations.

Recursive Dilated Convolutional Sequence: Unlike standard parallel architectures, SRDC adopts a recursive cascading structure where all three consecutive dilated convolutional layers strictly share a single, unified weight tensor $W_{share}\in R^{c^{\prime }\times c^{\prime }\times 3\times 3}$. By assigning progressively increasing dilation rates d∈{1,3,5} in a sequential loop, the module captures multi-granularity features continuously without compromising spatial resolution:

- Local Feature Level (Level 1, $d_{1}=1$): A standard 3×3 convolution is applied directly to $F_{base}$ to lock onto fine-grained spatial information, such as edges and textures:
  (7) $$Y_{1,k,i,j}=\sum_{c=0}^{c^{\prime }-1}\sum_{m=0}^{2}\sum_{n=0}^{2}W_{share,k,c,m,n}\cdot F_{base}\left(c,i+m-1,j+n-1\right)$$
- Extended Neighbour Level (Level 2, $d_{2}=3$): A moderate dilation rate is applied recursively to the output of the previous layer ($Y_{1}$). This captures associative features between the object and its immediate neighbourhood:
  (8) $$Y_{2,k,i,j}=\sum_{c=0}^{c^{\prime }-1}\sum_{m=0}^{2}\sum_{n=0}^{2}W_{share,k,c,m,n}\cdot Y_{1}\left(c,i+3\left(m-1\right),j+3\left(n-1\right)\right)$$
- Regional Semantic Level (Level 3, $d_{3}=5$): Wide-range regional cues are further aggregated by applying the shared kernel to the intermediate feature $Y_{2}$. This compensates robustly for the removal of the deep downsampling P5 layer:
  (9) $$Y_{3,k,i,j}=\sum_{c=0}^{c^{\prime }-1}\sum_{m=0}^{2}\sum_{n=0}^{2}W_{share,k,c,m,n}\cdot Y_{2}\left(c,i+5\left(m-1\right),j+5\left(n-1\right)\right)$$

In these expressions, the terms d(m−1) and d(n−1) explicitly represent the spatial offset induced by the dilation rate *d*. The selection of the tiered dilation sequence {1,3,5} is theoretically informed by the Hybrid Dilated Convolution framework to circumvent the “gridding effect” (i.e., checkerboard artifacts) prevalent in uniform dilated architectures [58]. Unlike standard dilation strategies that sample from sparse pixel grids and suffer from a loss of local continuity, this sequence of progressively increasing odd rates ensures that the effective receptive field expands consistently (from 3×3 to 7×7 and 11×11) while maintaining a dense sampling pattern. To guarantee that the spatial resolution remains strictly invariant throughout the sequence, adaptive padding p=d is maintained at each step. This fully convolutional paradigm facilitates a dynamic increase in the perceptual scope crucial for reconstructing the geometry of targets occupying only a few contiguous pixels while perfectly retaining the original h×w dimensions.

Feature Aggregation and Reconstruction: SRDC aggregates the original base feature $F_{base}$ with the cascaded outputs $\left\{Y_{1},Y_{2},Y_{3}\right\}$ along the channel dimension, yielding a concatenated tensor $X_{fusion}\in R^{4c^{\prime }\times h\times w}$. Consequently, this representation couples foundational spatial details with multi-granularity regional information. Finally, a 1×1 convolution performs channel alignment and fusion, producing the definitive output $X_{out}\in R^{c_{out}\times h\times w}$.

Theoretical Rationale of Weight Sharing: A potential theoretical concern is feature confusion specifically, whether applying the identical kernel $W_{share}$ to capture both highly localized textures (d=1) and broader semantics (d=5) degrades discriminative performance. However, this is elegantly avoided because SRDC employs a cascaded rather than a parallel structure. The input distribution shifts organically at each level ($F_{base}\to Y_{1}\to Y_{2}$). Consequently, the shared kernel is not applied repetitively to the raw spatial pixels at larger dilations, but rather to features that have already been progressively abstracted. In this context, the implementation of weight sharing serves primarily as an effective empirical design for extreme parameter efficiency and as a structural regularizer. It compels the network to learn a generalized, self-similar feature refinement transformation across tiered sampling intervals, preventing the network from overfitting to background noise.

Ultimately, by leveraging hierarchical dilation rates, SRDC establishes a tiered perceptual hierarchy uniquely suited to UAV imagery. While the low-dilation paths lock onto local textural fidelity, the high-dilation paths aggregate wide-area environmental logic. Consequently, SRDC markedly bolsters the representational capacity for infinitesimal object by seamlessly coupling foundational spatial cues with multi-granularity regional semantics, while circumventing the parameter explosion typical of standard multi-scale modules.

### 3.4. Deeply Decoupled Grouped Head (DGHead)

For UAV-based sensing tasks, leveraging high-resolution feature maps is vital for the successful identification of infinitesimal objects. To this end, this study incorporates the P2 feature layer (with 4× downsampling) into the YOLOv11n architecture, thereby enhancing the model’s feature perception capabilities regarding minute objects. Nevertheless, this modification imposes a significant computational burden.

**Figure 4:**
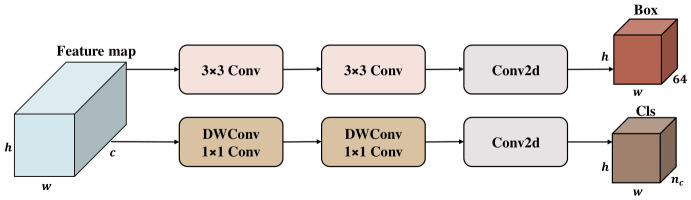
Structure of YOLOv11 Head.In the output tensors, the 64 channels in the Box branch correspond to the Distribution Focal Loss (DFL) representation, calculated as 4 bounding box boundaries (left, top, right, bottom) discretized into 16 bins (4×16=64). The $n_{c}$ channels in the Cls branch denote the classification probabilities for the total number of object classes.

Figure 4 depicts the deeply decoupled architecture employed by the original YOLOv11 detection head [55]. Specifically, the localisation branch stacks two layers of standard 3×3 convolutions, whereas the classification branch employs a combination of two sets of depthwise separable convolutions (DWConv) and 1×1 convolutions. Although this configuration enhances detection accuracy, the internal stacking of multiple 3×3 standard convolutions poses a challenge. Upon introducing the high-resolution P2 layer (with feature map dimensions of 160×160), the detection head’s floating-point operations (FLOPs) surge dramatically. This increase is due to the computational cost of standard 3×3 convolutions, which scale with the feature map resolution (h×w), severely constraining the model’s real-time inference capabilities.

**Figure 5:**
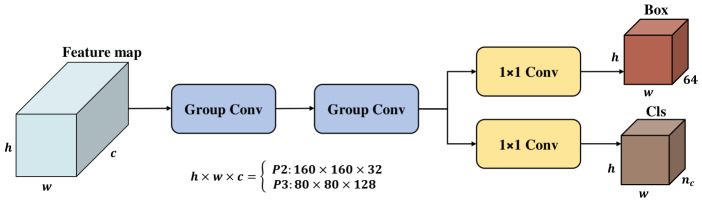
Structure of DGHead. The input dimensions (h×w×c) for the P2 and P3 feature layers are 160×160×32 and 80×80×128.

To achieve extreme lightweighting while maintaining the precision of the decoupled detection head, this paper proposes the Deeply Decoupled Grouped Head (DGHead). As depicted in Figure 5, the structural framework of the proposed head is composed of two main components: A task-decoupled prediction branch and a shared stem designed for grouped feature extraction.

Shared Grouped Feature Extraction Layer (Stem): The feature maps initially traverse two consecutive layers of 3×3 Grouped Convolutions (Group Conv). This design constitutes the core innovation of the DGHead; rather than employing standard convolutions for full-channel feature fusion, it leverages a grouping strategy to substantially mitigate computational overhead while preserving the depth of feature extraction. For an input feature map at the *i*-th scale, denoted as $X_{i}\in R^{c\times h\times w}$ (specifically the P2 and P3 layers in this model), the Stem layer yields an augmented feature representation, $F_{stem}$, which can be mathematically expressed through the following equation:

(10) $$F_{stem}={\mathrm{GConv}}_{3\times 3}\left({\mathrm{GConv}}_{3\times 3}\left(X_{i}\right)\right)$$

Task-Decoupled Prediction Branch: Upon acquiring the enhanced features $F_{stem}$, the network bifurcates into two parallel branches. The information flow diverges into dual task-specific pathways. In these branches, 1×1 kernels are used to estimate classification and localisation errors independently. This design ensures that the semantic features required for classification and the geometric features necessary for localisation are learned independently, thereby minimising inter-task interference.

**Figure 6:**
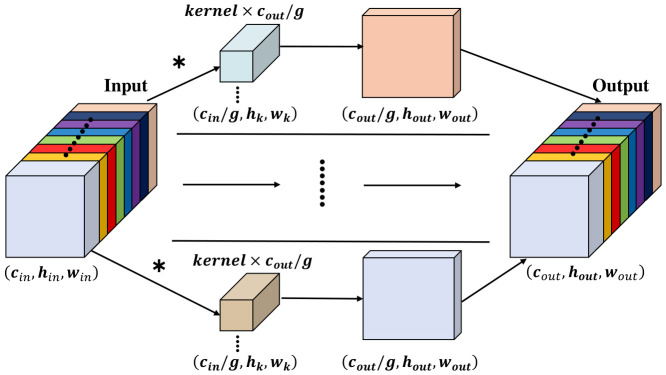
Structure of Group Conv. The asterisk (*) denotes the convolution operation. The ellipses (⋮ and …) represent the omitted intermediate groups. Different colors indicate the distinct groups into which the channels are divided.

The computational efficiency of the DGHead stems from its grouped convolution mechanism, as illustrated in Figure 6. The specific procedure is as follows: First, the $c_{in}$ channels of the input features are uniformly partitioned into *g* groups, with each group containing $c_{in}/g$ channels. Subsequently, convolution operations are performed independently within each group, generating $c_{out}/g$ output feature maps. Finally, the outputs from all groups are concatenated along the channel dimension to reconstruct the $c_{out}$ channels.

The efficiency of DGHead is evaluated quantitatively by comparing its overhead with that of traditional convolutions. this paper define $c_{in}$ and $c_{out}$ as the depths of input and output streams, while *k* signifies the kernel’s spatial, $h_{in}\times w_{in}$ indicate the dimensions of the feature map.

The resource requirements, in terms of parameter count ($P_{std}$) and computational load ($F_{std}$), for conventional convolutional layers are formulated as follows:

(11) $$P_{std}=k\times k\times c_{\mathrm{in}}\times c_{\mathrm{out}}=k^{2}c_{\mathrm{in}}c_{\mathrm{out}}$$

(12) $$F_{std}=h_{\mathrm{in}}\times w_{\mathrm{in}}\times c_{\mathrm{in}}\times c_{\mathrm{out}}\times k\times k=k^{2}h_{\mathrm{in}}w_{\mathrm{in}}c_{\mathrm{in}}c_{\mathrm{out}}$$

Conversely, for the Grouped Convolution (GConv) employed in the DGHead, the parameter count ($P_{GConv}$) and computational load ($F_{GConv}$) are expressed as:

(13) $$P_{GConv}=\left(\left(\frac{c_{\mathrm{in}}}{g}\right)\times \left(\frac{c_{\mathrm{out}}}{g}\right)\times k\times k\right)\times g=\frac{k^{2}c_{\mathrm{in}}c_{\mathrm{out}}}{g}$$

(14) $$F_{GConv}=h_{\mathrm{in}}\times w_{\mathrm{in}}\times \left(\frac{c_{\mathrm{in}}}{g}\right)\times \left(\frac{c_{\mathrm{out}}}{g}\right)\times k\times k\times g=\frac{k^{2}h_{\mathrm{in}}w_{\mathrm{in}}c_{\mathrm{in}}c_{\mathrm{out}}}{g}$$

Analytically, the implementation of grouped convolutions enables a theoretical reduction in both memory footprint and computational load by a factor of *g* relative to standard layers. Theoretically, while a larger *g* minimizes FLOPs, it risks creating a fragmented feature space where the sub-dimensionality of each group becomes insufficient to capture robust latent patterns. Conversely, a smaller *g* maintains rich channel synergy but incurs high structural redundancy. To reconcile these trade-offs and effectively mitigate the substantial computational burden introduced by the high-resolution P2 layer, this paper adopt a grouping constant of g=16 for the DGHead. The principled rationale for this specific value and its empirical impact on detection accuracy are discussed extensively in Section 4 (Experiments and Results). Through this architectural design, DGHead provides a viable, lightweight solution for real-time detection tasks in high-stakes UAV scenarios.

## 4. Experiments and Results

### 4.1. Dataset Introduction

**Figure 7:**
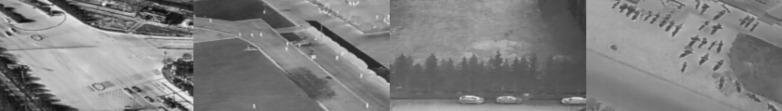
HIT-UAV dataset imagery.

**Figure 8:**
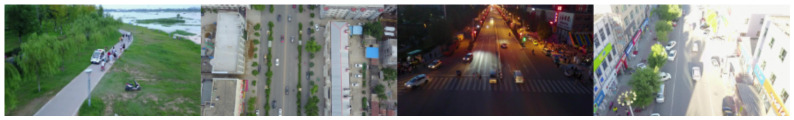
VisDrone2019 dataset imagery.

To rigorously validate LDSNet, this paper conducted evaluations on two prominent UAV benchmarks:

(1) HIT-UAV [59]: This dataset contains 2898 thermal images featuring low-altitude (30–60 m), infrared small object captured at 30–90° nadir angles. To optimize feature extraction, this paper consolidated “OtherVehicle” into “Car” and excluded “DontCare” samples, focusing on three core classes: Person, Car, and Bicycle. The data is partitioned into 2008 training, 571 validation, and 287 testing images (Figure 7).
(2) VisDrone2019 [56]: A comprehensive benchmark comprising high-resolution images (up to 2000×1500) captured across diverse urban landscapes, altitudes, and environmental conditions. It encompasses ten object categories and is divided into 6471 training, 548 validation, and 1610 testing images (Figure 8).

**Figure 9:**
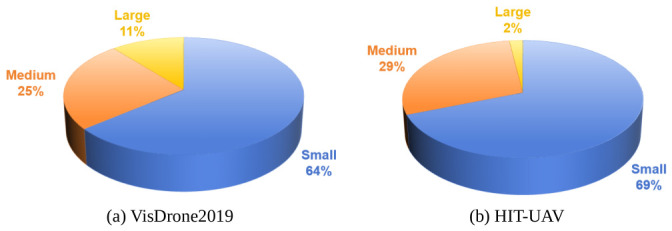
Distribution of object sizes for (**a**) VisDrone2019 and (**b**) HIT-UAV. Definition of scales (pixels): small (<$32^{2}$ pixels), medium ($32^{2}$ to $96^{2}$ pixels), and large (≥$96^{2}$ pixels).

The following Figure 9 illustrates the percentage distribution of large, medium, and small objects in the VisDrone2019 and HIT-UAV datasets.

### 4.2. Experimental Environment and Parameters

LDSNet was implemented, using the PyTorch framework on a Linux-powered workstation. For a rigorous and fair performance comparison, a consistent computational environment was maintained across all experimental trials, ensuring that LDSNet and all baseline architectures were evaluated under identical hardware and software constraints. Detailed system specifications are documented in Table 1.

The training hyperparameters are configured as follows, Table 2.

### 4.3. Evaluation Metrics

To rigorously assess detection accuracy and operational efficiency, this study utilises metrics spanning four dimensions: precision, spatial complexity, computational load, and inference velocity. Detection performance is primarily quantified through Precision (*P*), Recall (*R*), and Mean Average Precision (mAP), which are formulated as follows:

(15) $$P=\frac{TP}{TP+FP}\;,\;R=\frac{TP}{TP+FN}$$

In these definitions, TP, FP, and FN represent the counts of true positives, false positives, and false negatives, respectively. Furthermore, Average Precision (AP) is derived as the area under the Precision-Recall (P−R) curve, while mAP serves as the arithmetic mean of AP values across all *N* object categories:

(16) $$AP=\int_{0}^{1}P\left(r\right)dr\;,\;mAP=\frac{1}{N}\sum_{i=1}^{N}AP_{i}$$

Specifically, $mAP_{50}$ indicates the mean precision at an Intersection over Union (IoU) threshold of 0.5. To provide a more stringent evaluation of localisation robustness, $mAP_{50:95}$ is used to represent the average mAP over an IoU range from 0.5 to 0.95, with increments of 0.05.

Beyond accuracy, several indices are used to characterise the model’s resource utilisation and execution speed. The total number of trainable parameters, reported in millions (M), is used to measure the architecture’s spatial complexity and memory footprint. Computational intensity is quantified in terms of floating-point operations in billions (G), reflecting the processing load required for each forward pass. Lastly, the network’s real-time operational feasibility is assessed by the average inference time per frame, recorded in milliseconds (ms), which serves as a critical indicator of high-speed processing capability in practical UAV applications.

### 4.4. Structure Ablation

In response to the intrinsic challenges posed by the dense distribution and minute scale of small objects in UAV aerial imagery, this paper conducted a rigorous investigation into the combinations of Feature Pyramid levels (*S*) and backbone downsampling depths (*D*), as detailed in Table 3.

Empirical evidence indicates that the high-resolution P2 layer (4× downsampling) is vital for mitigating “semantic dissipation” often suffered by small objects in deep neural networks. By trading increased computational load for enhanced spatial granularity, this design preserves fine-grained geometric cues that deep layers typically annihilate. Notably, all architectural variants encompassing the P2 layer (e.g., *S*_234_-*D*_32×_) yielded ${\mathrm{mAP}}_{50}$ improvements ranging from 1.8% to 2.8% over the baseline, substantiating that the substantial accuracy gains justify the added FLOPs.

Beyond resolution considerations, analysis of downsampling depths reveals that the deep P5 layer is largely redundant for tiny UAV targets. Discarding P5 optimizes the resource budget, slashing parameter volume by 61.1% while incurring only a marginal ${\mathrm{mAP}}_{50}$ decline of 0.6% (dropping from 29.6% to 29.0%). This finding suggests that for low-altitude perspectives, computational capacity is more effectively utilized in high-resolution shallow paths than in overly abstracted deep features.

The determination of the final architecture follows the principle of diminishing returns: A comparison between *S*_234_-*D*_16×_ and *S*_23_-*D*_16×_ indicates that the P4 head offers a nominal ${\mathrm{mAP}}_{50}$ gain of only 0.1% at a disproportionate 10% FLOPs penalty (9.1 G to 10.0 G). To prioritize architectural economy, the *S*_23_-*D*_16×_ configuration was finalized as the optimal framework, hereafter designated as YOLOv11n-lite. This architecture achieves an ${\mathrm{mAP}}_{50}$ advantage of 1.8% over the original YOLOv11n while reducing parameter volume by 61.1%, effectively striking an accuracy-efficiency equilibrium by concentrating processing power on high-gain shallow features. It thus serves as the streamlined baseline for integrating subsequent modular innovations, including LDSDown, SRDC, and DGHead.

### 4.5. LDSDown Evaluation

Downsampling is a vital mechanism for modulating the receptive fields and spatial resolutions of feature maps. To assess the performance of LDSDown, this paper conducted comparative benchmarks against established methods such as Haar Wavelet Downsampling (HWD) [17], SPDDown [16], V7DS [60] and GCDown [61]. The quantitative performance metrics are detailed in Table 4.

Experimental data indicate that LDSDown achieves an optimal balance in architectural lightweighting. In terms of computational cost, LDSDown reduces FLOPs by 9.9% (from 9.1G to 8.2G) and parameter count by 28.6% (from 0.7M to 0.5M). The design rationale behind this efficiency stems from the channel-splitting strategy, which effectively eliminates redundant inter-channel computations that typically occur in standard convolutions. Although HWD achieves a similar parameter count, its higher computational demand (8.4GFLOPs) suggests that LDSDown’s parallel extraction is more adept at targeting spatial redundancy than frequency-domain transformations.

While the SPDDown module yields the highest accuracy (${\mathrm{mAP}}_{50}=29.7\%$), it imposes a prohibitive computational burden (12.6G), which is nearly double that of LDSDown. For resource-constrained UAV platforms, this reflects a sub-optimal trade-off where the accuracy gain does not justify the massive resource overhead. Conversely, LDSDown maintains a high “accuracy-per-parameter” ratio. Despite a significant reduction in model size, it incurs only a negligible 0.5% drop in ${\mathrm{mAP}}_{50}$. Crucially, the anti-aliasing phase in LDSDown serves as an information filter, suppressing sampling noise that would otherwise lead to false positives. This is evidenced by the improvement in Precision (39.8% vs. 39.6%), confirming that the coupling of textural enhancement with saliency compensation allows the network to extract high-quality features while strictly limiting its computational footprint. Compared to V7DS and GCDown, LDSDown offers a more principled solution for high-throughput UAV sensing by prioritizing feature purity over raw parametric depth.

### 4.6. SRDC Evaluation

**Figure 10:**
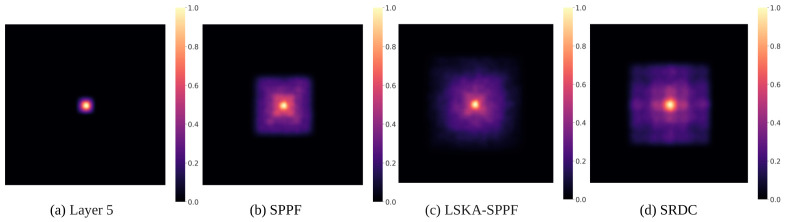
Comparative visualisation of effective receptive field: (**a**) Highly concentrated ERF of Layer 5; (**b**) Centre-biased response of SPPF; (**c**) Expanded coverage of LSKA-SPPF; (**d**) Radiative outward expansion of the proposed SRDC module.

The effective receptive field (ERF) is a primary indicator of how well a network exploits long-range spatial context [62]. Our investigation into the Shared Recursive Dilated Convolution (SRDC) module included both ERF saliency analysis (Figure 10) and performance trials against other SPPF modifications.

Figure 10a (Layer 5): The baseline receptive field is highly concentrated and limited in scope, failing to encompass the environmental context surrounding the object.

Figure 10b (SPPF): While the original SPPF expands the field via pooling, the energy distribution remains centre-focused with weak edge responses, limiting the modelling of large-scale backgrounds in complex scenarios.

Figure 10c (LSKA-SPPF) [19]: The introduction of Large Selective Kernel Attention (LSKA) expands coverage significantly.

Figure 10d (SRDC): The proposed SRDC exhibits the most extensive ERF, characterised by a radiative outward expansion. This is attributed to the recursive cascade design, where dilated convolutions with increasing rates (d=1,3,5) propagate features hierarchically. This induces an exponential spatial growth in the receptive field, enhancing the perception of long-range semantic information and mitigating the “semantic dissipation” of small object common in UAV aerial views.

As shown in Table 5, SRDC outperforms mainstream improvements (AIFI [18], FMSPPF [63], LSKA-SPPF) under identical conditions. It achieves the most competitive results, reaching a ${\mathrm{mAP}}_{50}$ of 29.9%. To further isolate the impact of the weight-sharing mechanism, this paper evaluated a variant with independent weights, denoted as RDC. The results show that while RDC marginally improves ${\mathrm{mAP}}_{50}$ to 30.1%, it nearly doubles the module’s parameter count and quadruples its computational load. Most importantly, despite its higher parametric capacity, RDC yields lower Precision than SRDC (40.8% vs. 41.5%). This discrepancy validates our theoretical hypothesis: the weight-sharing mechanism in SRDC does not merely compress the model, but also functions as a structural regularizer. By compelling the same kernels to perceive features across tiered dilation rates, SRDC promotes the learning of scale-invariant geometric patterns and suppresses background noise. Consequently, SRDC provides a more robust representation of minute object while offering a significantly more optimised trade-off between detection performance and model complexity.

### 4.7. DGHead Evaluation

To evaluate the impact of the grouping factor *g* on detection efficiency, this paper conducted a parametric sweep (Table 6). This design choice fundamentally governs the trade-off between inter-channel feature synergy and computational sparsity. Although the 1/g relationship theoretically suggests continuous resource reduction, empirical data reveal that practical computational overhead increases at higher group counts, likely due to implementation-level memory access fragmentation. At low *g* values, the restricted channel grouping fails to extract sufficient cross-channel information, resulting in a significant decline in mAP. However, representational capacity recovers as *g* increases, reaching an optimal equilibrium at g=16. At this configuration, the model maintains sufficient representational granularity to nearly match baseline accuracy (${\mathrm{mAP}}_{50}=28.8\%$) while drastically slashing FLOPs by 39.6%. Further increasing *g* to 32 yields negligible precision gains but incurs higher computational costs, confirming g=16 as the principled “sweet spot” for balancing representational power with operational economy.

This paper benchmarked DGHead against the native YOLOv11 head and other improved variants (LADH [64], LQE [65], LSCD [66], SEAM [67]). As shown in Table 7, DGHead demonstrates superior efficiency, reducing computation to 5.5 G FLOPs (a 39.6% reduction vs. baseline) and parameters to 0.5 M. Despite this extreme lightweighting, it maintains competitive accuracy (${mAP}_{50:95}=16.1\%$), on par with the original head and superior to LADH (15.5%). Additionally, DGHead achieves the highest Precision (40.2%) among all compared methods. Compared to high-precision heads like LQE, DGHead sacrifices only 0.5% in ${mAP}_{50}$ but achieves nearly 40% in computational savings, effectively balancing the high computational pressure introduced by high-resolution features.

### 4.8. Overall Ablation

To verify the synergistic benefits of LDSNet, a comprehensive ablation study was performed on the VisDrone2019 dataset using YOLOv11n as a reference (Table 8). The results demonstrate how each module contributes to a superior accuracy-efficiency equilibrium through strategic resource reallocation.

Initially, the structural reconfiguration of the baseline (YOLOv11n-lite) demonstrated a significant performance gain, with ${\mathrm{mAP}}_{50}$ increasing from 27.2% to 29.0% and Recall reaching a peak of 31.5%. This result confirms that integrating the high-resolution P2 feature layer is essential for capturing the minute spatial details of small object. Notably, although the increased resolution led to an expected rise in FLOPs (to 9.1 G), the total parameter count dropped substantially from 2.6 M to 0.7 M, establishing a solid foundation for further lightweight optimisation.

LDSDown contributes to spatial integrity and initial lightweighting. It refines the high-resolution flow by filtering sampling noise, further reducing parameters to 0.5 M and FLOPs to 8.2 G. This proves that our dual-path strategy is more cost-effective than standard downsampling, preserving fragile features at a lower computational price.Subsequently, the integration of DGHead massively reduced the computational redundancy, plunging the FLOPs down to an astonishing 4.6 G and parameters to 0.4 M. This indicates that its decoupled and grouped structure effectively minimises the computational burden between classification and localisation tasks, albeit with a slight, acceptable trade-off in temporary ${\mathrm{mAP}}_{50}$ fluctuation.

Finally, the incorporation of the SRDC module catalysed a substantial performance rebound, elevating the ${\mathrm{mAP}}_{50}$ from 28.2% to 29.4% and Precision from 39.7% to 41.1%, remarkably without incurring any additional parameter or computational overhead (remaining at 0.4 M and 4.6 G FLOPs, respectively). This improvement validates the strategy of replacing deterministic pooling with cascaded dilated convolutions, as it preserves fine-grained spatial information while effectively expanding the receptive field to extract multi-scale regional features from complex backgrounds.

Ultimately, the fully integrated LDSNet achieves a superior equilibrium between detection fidelity and computational efficiency. Compared to the original baseline, LDSNet yields a 2.2% improvement in ${\mathrm{mAP}}_{50}$ (reaching 29.4%) and a 2.5% increase in Precision (reaching 41.1%), while simultaneously reducing the computational load by approximately 29.2% (from 6.5 G to 4.6 G FLOPs) and parameter volume by 84.6% (from 2.6 M to 0.4 M). These outcomes demonstrate that the synergistic combination of high-resolution feature capture, efficient grouped convolutions, and tiered regional perception ensures robust performance for demanding UAV-based remote sensing tasks.

### 4.9. Model Comparison

To ensure a rigorous evaluation, LDSNet was benchmarked against diverse modern detectors under identical hardware and training protocols. These comparative models include mainstream one-stage models (YOLOv5n to YOLOv10n [68,69,70], and YOLOv26n [71]), efficient YOLO variants (ITD-YOLOv8 [72], G-YOLO [73], YOLOv5n+TDAM [74]), UAV-specific lightweight architectures (LRI-YOLO [75], ELNet [5], DLNet [76], Drone-YOLO [77]), and the transformer-based RT-DETR [78].

As shown in Table 9, LDSNet effectively balances accuracy and efficiency on the HIT-UAV infrared dataset. It achieves an ${\mathrm{mAP}}_{50}$ of 94.5%, outperforming the YOLOv11n baseline (93.3%) and RT-DETR (93.0%). Furthermore, LDSNet reaches a peak ${\mathrm{mAP}}_{50:95}$ of 62.0%, demonstrating superior localization precision in complex thermal backgrounds. Structurally, LDSNet maintains an exceptionally compact footprint, with a parameter volume of only 0.4 M and a computational cost of 4.6 G, representing an 84.6% reduction in parameters compared to YOLOv11n. Although marginally larger than ELNet (0.3 M), LDSNet’s substantial gains in detection accuracy and robustness easily justify this minimal overhead.

The VisDrone2019 dataset, characterised by dense clusters of minute object, poses even greater challenges for feature extraction. According to the results summarized in Table 10, LDSNet attained an ${\mathrm{mAP}}_{50}$ of 29.4%, which outstrips the YOLOv11n baseline by 2.2%, and exceeds both YOLOv12n (27.1%) [79] and the lightweight ELNet (28.4%). Furthermore, LDSNet achieves a Precision of 41.1%, the highest among all lightweight models with fewer than 3 M parameters, demonstrating its ability to suppress false positives arising from background clutter effectively. In terms of real-time performance, the single-frame inference latency of LDSNet is 1.8 ms, which is comparable to other lightweight YOLO variants and significantly faster than RT-DETR (21.3 ms).

Synthesising the results from both benchmarks, LDSNet successfully fulfils its intended design objectives. The integration of LDSDown and DGHead significantly reduces computational and parametric complexity, effectively neutralising the overhead typically incurred by high-resolution feature layers such as P2. Simultaneously, the SRDC module mitigates the information loss inherent in traditional downsampling by expanding the effective receptive field and bolstering hierarchical regional associations. Consequently, LDSNet offers a robust solution for UAV remote sensing, delivering superior detection fidelity with a minimal computational footprint compared to mainstream lightweight alternatives.

Beyond static image benchmarks, the operational potential of LDSNet in dynamic video sequences is a critical factor for real-life UAV applications such as traffic surveillance and disaster response. As demonstrated in Table 9 and Table 10, the raw inference latency of LDSNet is 1.6–1.8 ms per frame on an NVIDIA RTX 3090 GPU. In a practical end-to-end video processing pipeline, which encompasses image pre-processing (resizing and normalization) and post-processing (Non-Maximum Suppression, NMS), the total latency remains highly competitive.

Given that LDSNet maintains a similar computational profile to the YOLOv11n baseline, its total end-to-end latency is estimated to be well within the range required for high-speed real-time processing (>100 FPS). According to recent studies on real-time UAV detection [28,81], a frame rate of 30–60 FPS is typically sufficient for standard monitoring, whereas LDSNet offers a significant performance margin that can accommodate higher-resolution streams or multi-target tracking algorithms without incurring accumulation delay. The architectural economy of LDSNet (0.4 M Params) further reduces the likelihood of “tail latency” during complex scene transitions in dynamic videos, ensuring temporal consistency for localized bounding boxes. These factors collectively indicate that LDSNet possesses robust potential for low-latency, real-time deployment in high-stakes dynamic aerial environments.

### 4.10. Scale-Wise Robustness Evaluation

To quantitatively evaluate the robustness of LDSNet across varying object dimensions, this paper conducted a comparative analysis using the standard COCO evaluation protocol [82], categorizing targets into small, medium, and large scales. As summarized in Table 11, LDSNet demonstrates a significant performance advantage in the categories most relevant to UAV perspectives. Specifically, for the AP-Small metric, LDSNet achieves 7.4%, outperforming YOLOv11n and YOLOv12n by 1.6% and 1.7% respectively, which corresponds to a relative improvement of over 27%. A similar trend is observed in the AP-Medium category, where LDSNet reaches 24.4%. While the baseline models show a marginal lead in the AP-Large indicator, this is a reasonable trade-off given that LDSNet is specifically re-engineered to prioritize “pixel-poor” infinitesimal targets by sacrificing the redundant deep-layer receptive fields. These results substantiate that LDSNet is highly optimized for UAV-captured scenarios where small and medium-scale objects predominate.

### 4.11. Visual Analysis

**Figure 11:**
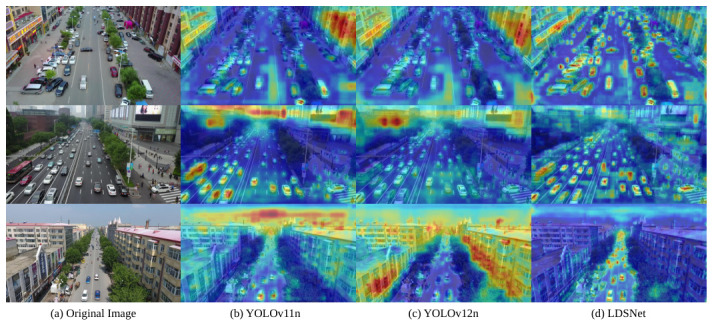
Grad-CAM heatmap visualisation and comparative analysis across different detection models: (**a**) Original aerial images; (**b**) YOLOv11n baseline; (**c**) YOLOv12n baseline; and (**d**) the proposed LDSNet. Compared to the baselines in (**b**,**c**), the proposed LDSNet in (**d**) exhibits more concentrated and precise feature activation on minute object while significantly suppressing non-object background clutter. The colors in the heatmaps indicate the intensity of feature activation: warmer colors (e.g., red) represent regions with high activation where the model’s attention is primarily focused, while cooler colors (e.g., blue) correspond to low-activation background regions.

The model’s feature extraction and localisation prowess were visualised using Grad-CAM [83]. By contrasting LDSNet with baseline architectures, this paper demonstrate its superior capacity to perceive dense clusters in high-complexity scenarios (Figure 11). The analysis reveals that LDSNet produces significantly more concentrated and precise feature responses. Heatmaps generated by the baseline models exhibit diffuse distributions, with spatial attention frequently spilling over into non-object regions, such as rooftops, vegetation, and road surfaces. Notably, in highway scenarios with high vehicular density, the baselines tend to merge the feature responses of adjacent vehicles into continuous, large-scale saliency regions. This inability to delineate individual boundaries reflects a deficiency in feature discriminability and a confusion of regional cues when processing dense, minute object. Conversely, the high-activation regions of LDSNet (indicated in red) align precisely with the object contours. Even for distant vehicles with minimal pixel occupancy, LDSNet consistently generates distinct, isolated feature response points. Furthermore, the model demonstrates superior background suppression. This performance gain is primarily attributed to the SRDC module, which utilises tiered dilation rates to establish hierarchical spatial associations. By cross-referencing fine-grained details with regional environmental logic, the model effectively distinguishes genuine object from background clutter, significantly enhancing the discriminability of feature representations.

**Figure 12:**
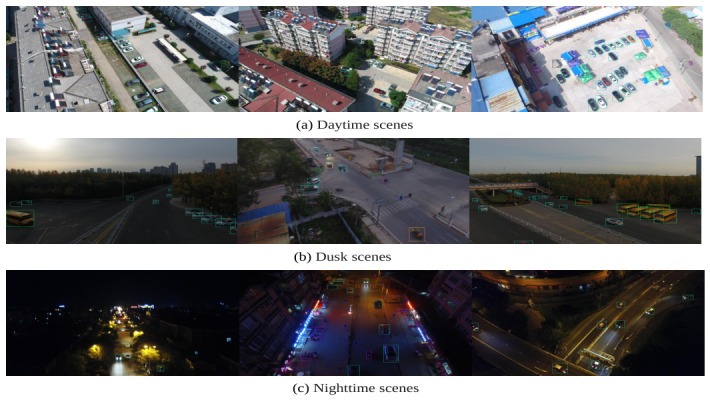
Qualitative detection results of LDSNet under diverse illumination conditions on the VisDrone2019 dataset: (**a**) daytime scenes; (**b**) dusk scenes; and (**c**) nighttime scenes. The results underscore the model’s robustness and its ability to maintain stable detection performance across significant temporal and lighting fluctuations. The colored frames represent the predicted bounding boxes, indicating the precise locations and categories of the detected objects.

To further substantiate detection performance and small-object acquisition capability, this paper conducted a comprehensive qualitative analysis across a range of precisely defined, intricate scenarios. As illustrated in Figure 12, LDSNet maintains consistent detection capabilities across diverse illumination conditions, including daylight, dusk, and nighttime. This demonstrates the model’s resilience against imaging quality degradation often caused by drastic lighting variations, ensuring stable performance in round-the-clock surveillance tasks.

**Figure 13:**
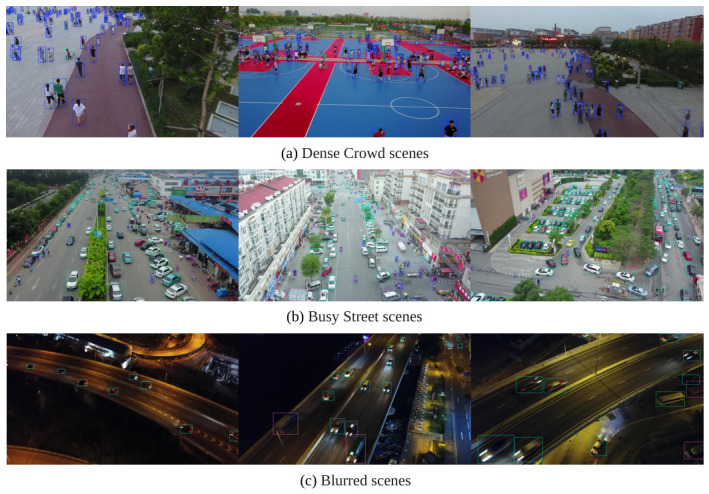
Detection performance of LDSNet in representative high-challenge aerial environments: (**a**) dense crowd scenes; (**b**) busy street scenes; and (**c**) blurred imagery. The visualisations demonstrate that LDSNet preserves high detection integrity and localisation veracity despite cluttered backgrounds and motion-induced degradation. The colored frames represent the predicted bounding boxes, indicating the precise locations and categories of the detected objects.

Furthermore, the model’s robustness is evidenced in high-challenge environments such as dense crowds, heavy urban traffic, and motion-blurred scenes, as shown in Figure 13. The visualisations confirm that LDSNet maintains high detection integrity and localisation accuracy even under these adverse conditions, where the proximity of object and the lack of clear boundaries typically hinder conventional detectors.

**Figure 14:**
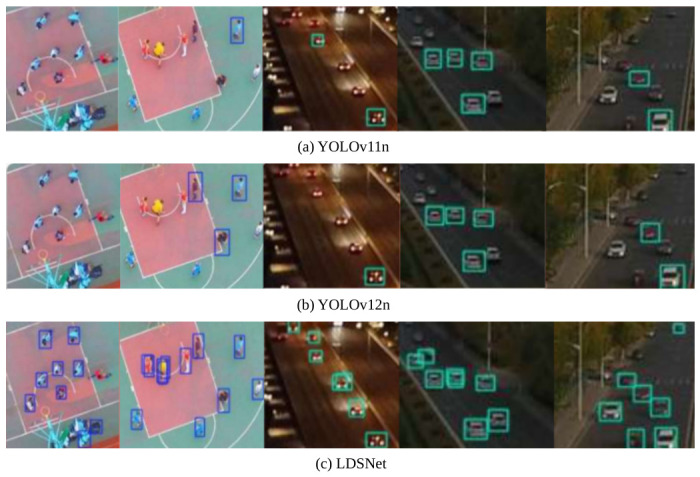
Comparative visualisation of detection performance on clustered minute object: (**a**) YOLOv11n; (**b**) YOLOv12n; and (**c**) the proposed LDSNet. The red magnified insets highlight LDSNet’s superior recall, successfully pinpointing infinitesimal objects that are frequently missed by standard baselines. The colored frames represent the predicted bounding boxes, indicating the precise locations and categories of the detected objects. The colored frames represent the predicted bounding boxes, indicating the precise locations and categories of the detected objects.

Addressing one of the most critical hurdles in UAV imagery, Figure 14 compares the performance of YOLOv11n, YOLOv12n, and LDSNet on clustered minute targets. The red magnified sub-views reveal that baseline models frequently miss detections when targets have extremely low pixel occupancy. In contrast, LDSNet successfully resolves these individual targets, which often span only a few pixels, validating the effectiveness of the proposed Detail-Sensitive mechanism.

**Figure 15:**
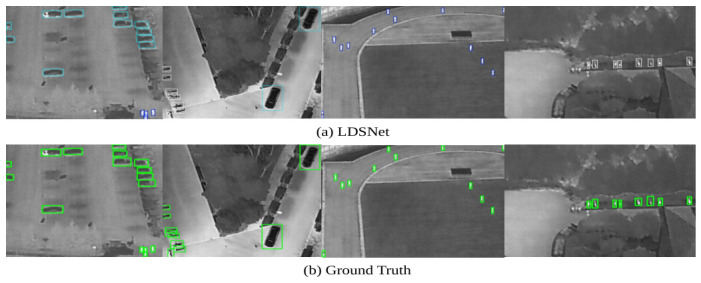
Visualisation of detection results versus ground-truth annotations on the HIT-UAV infrared dataset: (**a**) LDSNet predictions; and (**b**) ground truth labels. The model achieves high-fidelity alignment and effective edge preservation, showcasing its sensitivity even in the absence of chromatic and textural cues. The colored frames represent the bounding boxes of the objects. Specifically, the cyan frames in (**a**) denote the detection results predicted by LDSNet, while the green frames in (**b**) indicate the ground-truth bounding boxes of the actual targets.

To verify efficacy across data modalities, further evaluations were performed on the HIT-UAV infrared dataset. As shown in Figure 15, despite the inherent lack of textural information and chromatic cues in thermal imagery, the bounding boxes generated by LDSNet align meticulously with the ground truth. This suggests that the adaptive feature enhancement mechanism exhibits exceptional sensitivity and edge-preservation even in the infrared domain.

**Figure 16:**
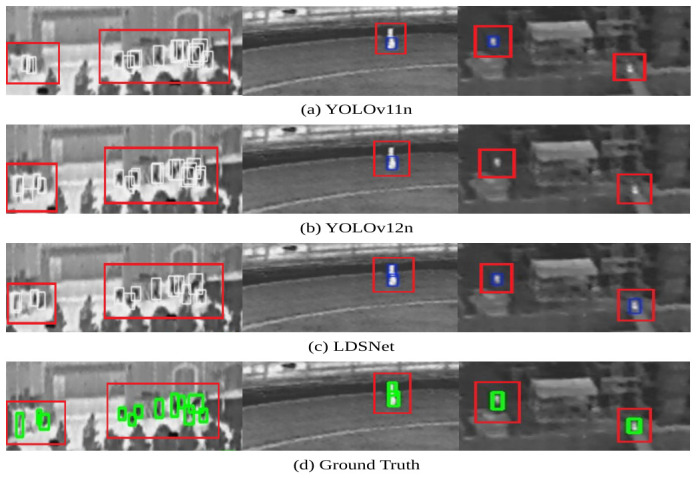
Detailed detection performance comparison for dense and occluded infrared object: (**a**) YOLOv11n; (**b**) YOLOv12n; (**c**) the proposed LDSNet; and (**d**) ground truth labels. Compared to baselines, LDSNet exhibits greater discriminability and superior alignment in complex thermal backgrounds. The colored frames represent the bounding boxes of the objects. Specifically, the white and blue frames in (**a**–**c**) denote the detection results predicted by the respective models, while the green frames in (**d**) indicate the ground-truth bounding boxes. The prominent red rectangles are utilized to highlight specific comparison regions, particularly showcasing instances where the baseline models (YOLOv11n and YOLOv12n) fail to detect the targets.

A comparative analysis of these thermal results, provided in Figure 16, indicates that baseline models exhibit a high frequency of redundant bounding boxes and false negatives in regions containing dense infrared object, often identifying only the most prominent objects. Conversely, LDSNet demonstrates significantly higher fidelity to the ground truth. Even in scenarios involving occluded or blurred tiny infrared object, where baselines show insufficient sensitivity, LDSNet’s detections remain in close agreement with the ground truth, effectively covering nearly all visible objects while suppressing thermal background noise.

**Figure 17:**
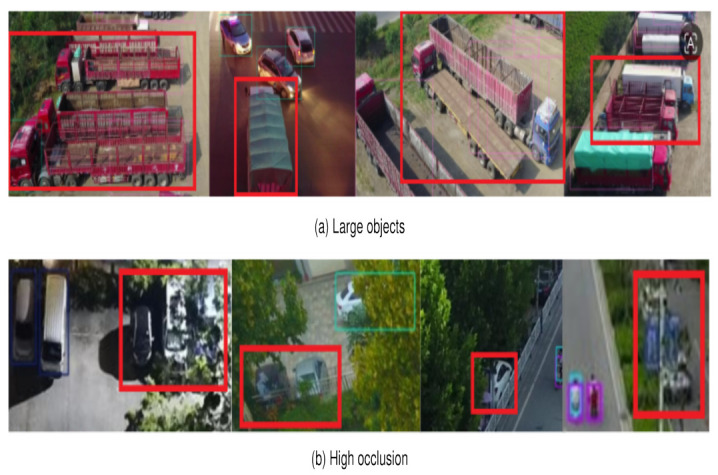
Visualization of typical failure conditions for the proposed method: (**a**) Large objects: the restricted downsampling depth limits the global receptive field, resulting in mismatched spatial coverage and localization inaccuracy. (**b**) High occlusion: severe inter-object overlap and background obstruction lead to the loss of critical features, causing missed detections. The thin colored frames represent the predicted bounding boxes of the detected objects. The prominent red rectangles are specifically utilized to highlight the failure cases of the proposed LDSNet, such as inaccurate localization or missed detections in these challenging scenarios.

Beyond the robust performance demonstrated on minute targets, certain failure cases were observed in specific challenging scenarios. As illustrated in Figure 17, LDSNet exhibits suboptimal efficacy when encountering large-scale objects or extreme occlusion. In the case of large objects (Figure 17a), the model occasionally suffers from localization inaccuracies or fragmented detections. This limitation primarily stems from the architectural decision to remove the deep P5 layer and restrict downsampling to 16×, a strategy that prioritizes fine-grained details but inherently constricts the global receptive field needed for the holistic modeling of large entities. Furthermore, detections often fail in scenarios characterized by high occlusion (Figure 17b). When targets are severely overlapped or obstructed by background elements, their already sparse feature representations are further diminished, making it difficult for the model to resolve target-background ambiguity. These observations highlight the model’s current limitations in handling multi-scale variance and physical shielding, suggesting that future research could focus on incorporating adaptive receptive field mechanisms or exploring temporal information to enhance robustness in cluttered environments.

## 5. Conclusions

This paper introduce LDSNet, an efficient detection framework specifically engineered to identify tiny objects within low-altitude UAV imagery. Tailored to the unique scale and distribution of aerial object, the feature pyramid was restructured by omitting the deep P5 layer (with 32× downsampling) to avert semantic annihilation. In contrast, a high-resolution P2 layer (with 4× downsampling) was integrated to bolster detail capture. LDSNet incorporates three primary architectural innovations: LDSDown integrates anti-aliasing preprocessing with a dual-path strategy to safeguard spatial integrity; SRDC establishes hierarchical regional associations via recursive weight-sharing and tiered dilation rates without parameter inflation; and DGHead linearises the computational overhead of high-resolution features using grouped convolutions.

Quantitative evaluations confirm that LDSNet achieves a good balance between detection accuracy and computational efficiency. Specifically, on the VisDrone2019 benchmark, the model achieved a 29.4% ${\mathrm{mAP}}_{50}$ (exceeding YOLOv11n by 2.2%) with a 1.8 ms inference latency. This was accomplished alongside an 84.6% reduction in parameter volume and a 29.2% decrease in computational complexity. Furthermore, trials on the HIT-UAV infrared dataset yielded a superior ${\mathrm{mAP}}_{50}$ of 94.5%, surpassing contemporary architectures like RT-DETR. Visual interpretability further validates the model’s ability to localise and robustly suppress background accurately.

Despite its robust performance on minute targets, LDSNet involves certain architectural trade-offs. The strategic omission of the P5 layer, while optimizing detail sensitivity, inherently constricts the global receptive field. Consequently, the model may exhibit suboptimal efficacy when encountering large-scale objects or extreme occlusion, where sparse feature representations are further diminished by physical shielding. Nevertheless, the core design logic of LDSNet remains highly generalizable to broader air-to-ground observation scenarios across the remote sensing community, such as satellite monitoring and maritime surveillance, where resolving the fundamental bottlenecks of small object detection is critical. While the current framework is specialized for minute targets, its potential applicability to general object detection domains involving large-scale natural images remains a subject for future investigation, where adaptive receptive field scaling could be further explored to bridge the gap in multi-scale representation.

Subsequent investigations will prioritise two primary trajectories. First, this paper intends to validate execution efficiency on embedded platforms such as Jetson and FPGA by employing hardware-aware strategies, including INT8 quantisation, to further optimise real-time flight performance. Second, this paper plans to extend the architecture to facilitate the fusion of visible-light, thermal, and LiDAR data, thereby significantly enhancing detection reliability and robustness under adverse meteorological conditions.

## Figures and Tables

**Table 1:** Experimental environment configuration.

Component	Configuration
Operating System	Ubuntu 20.04
GPU	NVIDIA GeForce RTX 3090 (24 GB)
CPU	Intel(R) Xeon(R) Platinum 8375C CPU @ 2.90 GHz
Python Version	3.10.14
Deep Learning Framework	PyTorch 2.4.0
CUDA Version	12.4

**Table 2:** Hyperparameter configurations for model training.

Name	Value	Name	Value
Optimizer	SGD	Training Epochs	300
Input Image Size	640×640	Data Loading Workers	4
Initial Learning Rate	0.01	Batch Size	16
Early Stopping	Enabled	Automatic Mixed Precision (AMP)	Enabled
Weight Decay	0.0005	Momentum Factor	0.937

**Table 3:** Deep ablation analysis of detection performance with various network structure adjustments.

Scheme (*S*-*D*)	P2	P3	P4	P5	Conv5	P (%)	R (%)	${\mathrm{mAP}}_{50}$ (%)	${\mathrm{mAP}}_{50:95}$ (%)	Param (M)	FLOPs (G)
*S*_345_-*D*_32×_ (Baseline)		✓	✓	✓	✓	38.6	29.4	27.2	15.1	2.6	6.5
*S*_34_-*D*_16×_ (Minimal)		✓	✓			37.9	28.9	25.6	14.3	**0.7**	**4.6**
*S*_34_-*D*_16×_ (Standard)		✓	✓		✓	38.6	31.1	27.4	15.2	1.8	5.7
*S*_234_-*D*_16×_ (Lite)	✓	✓	✓			39.8	30.7	29.1	16.4	0.9	10.0
*S*_234_-*D*_16×_ (Full)	✓	✓	✓		✓	40.4	**32.2**	**30.0**	**16.8**	2.1	11.1
*S*_23_-*D*_16×_ (Lite)	✓	✓				39.6	31.5	29.0	16.1	**0.7**	9.1
*S*_23_-*D*_16×_ (Full)	✓	✓			✓	**40.7**	31.8	29.6	16.5	1.8	10.2

Note: Scale Levels (*S*) represents the ensemble of feature hierarchies participating in detection (e.g., $S_{234}$ utilizes P2, P3, P4 layers); Depth Configuration (*D*) refers to the backbone subsampling depth ($D_{32\times }$ retains the P5 layer. In contrast, $D_{16\times }$ restricts downsampling to 16×). Conv5 indicates the inclusion of the P5-specific convolutional block. Precision (*P*), Recall (*R*), and mAP are measured in percentage (%). Bold values denote the best performance in each metric. Param and FLOPs are quantified in Millions (M) and Giga (G), respectively. The checkmark (✓) denotes the inclusion of the corresponding feature layer or module in the specific scheme. Bold values denote the best performance in each metric.

**Table 4:** Comparison of different downsampling modules based on YOLOv11n-lite.

Methods	P (%)	R (%)	${\mathrm{mAP}}_{50}$ (%)	${\mathrm{mAP}}_{50:95}$ (%)	Param (M)	FLOPs (G)
YOLOv11n-lite	39.6	**31.5**	29.0	16.1	0.7	9.1
YOLOv11n-lite-LDSDown	39.8	30.7	28.5	15.8	**0.5**	**8.2**
YOLOv11n-lite-HWD	39.1	30.3	28.2	15.6	**0.5**	8.4
YOLOv11n-lite-V7DS	40.3	31.2	28.7	16.1	0.6	8.8
YOLOv11n-lite-SPDDown	**41.4**	31.4	**29.7**	**16.6**	1.3	12.6
YOLOv11n-lite-GCDown	40.1	31.4	29.0	16.4	0.7	9.1

Note: Bold values denote the best performance in each metric.

**Table 5:** Comparison of different SPPF modules based on YOLOv11n-lite.

Methods	P (%)	R (%)	${\mathrm{mAP}}_{50}$ (%)	${\mathrm{mAP}}_{50:95}$ (%)	Param (M)	FLOPs (G)
YOLOv11n-lite-SPPF	39.6	31.5	29.0	16.1	0.7	**9.1**
YOLOv11n-lite-SRDC	**41.5**	31.6	29.9	16.9	**0.7**	**9.1**
YOLOv11n-lite-RDC	40.8	**31.8**	**30.1**	**17.0**	0.8	9.4
YOLOv11n-lite-AIFI	39.9	30.7	29.2	16.4	0.8	9.3
YOLOv11n-lite-FMSPPF	40.3	30.9	29.1	16.4	0.7	**9.1**
YOLOv11n-lite-LSKA-SPPF	40.9	31.7	29.7	16.7	0.7	9.3

Note: RDC denotes the version of our module without weight sharing. Bold values denote the best performance in each metric.

**Table 6:** Sensitivity analysis of the grouping factor *g* in DGHead.

Methods	P (%)	R (%)	${\mathrm{mAP}}_{50}$ (%)	${\mathrm{mAP}}_{50:95}$ (%)	Param (M)	FLOPs (G)
YOLOv11n-lite	39.6	**31.5**	**29.0**	16.1	0.7	9.1
YOLOv11n-lite-DGHead (g=2)	39.3	30.2	28.0	15.4	**0.5**	**4.7**
YOLOv11n-lite-DGHead (g=4)	39.5	29.9	28.1	15.7	**0.5**	4.8
YOLOv11n-lite-DGHead (g=8)	39.8	30.4	28.4	15.8	**0.5**	5.0
YOLOv11n-lite-DGHead (g=16)	40.2	30.8	28.8	16.1	**0.5**	5.5
YOLOv11n-lite-DGHead (g=32)	**40.3**	31.3	28.8	**16.2**	0.6	6.4

Note: Bold values denote the best performance in each metric.

**Table 7:** Comparison of detection performance with different detection heads based on YOLOv11n-lite.

Methods	P (%)	R (%)	${\mathrm{mAP}}_{50}$ (%)	${\mathrm{mAP}}_{50:95}$ (%)	Param (M)	FLOPs (G)
YOLOv11n-lite	39.6	31.5	29.0	16.1	0.7	9.1
YOLOv11n-lite-DGHead	**40.2**	30.8	28.8	16.1	**0.5**	**5.5**
YOLOv11n-lite-LADH	38.5	30.5	27.9	15.5	**0.5**	5.9
YOLOv11n-lite-LQE	40.1	**31.7**	**29.3**	**16.4**	0.7	9.1
YOLOv11n-lite-LSCD	39.6	30.9	28.8	16.1	0.6	6.8
YOLOv11n-lite-SEAM	38.6	31.0	28.4	15.7	0.6	7.0

Note: Bold values denote the best performance in each metric.

**Table 8:** Ablation study of the proposed LDSNet modules on the VisDrone2019 dataset.

yolov11n	Lite	LDSDown	DGHead	SRDC	P (%)	R (%)	${\mathrm{mAP}}_{50}$ (%)	${\mathrm{mAP}}_{50:95}$ (%)	Param (M)	FLOPs (G)
✓					38.6	29.4	27.2	15.1	2.6	6.5
✓	✓				39.6	**31.5**	29.0	16.1	0.7	9.1
✓	✓	✓			39.8	30.7	28.5	15.8	0.5	8.2
✓	✓	✓	✓		39.7	30.1	28.2	15.7	**0.4**	**4.6**
✓	✓	✓	✓	✓	**41.1**	30.9	**29.4**	**16.4**	**0.4**	**4.6**

Note: ✓ means that the module is activated. “Lite” refers to the reconstruction of the feature pyramid (introducing P2 and removing P5). Bold values indicate the best results in each metric. Param and FLOPs represent model size and computational complexity, respectively. Bold values denote the best performance in each metric.

**Table 9:** Performance comparison of various detectors on the HIT-UAV dataset.

Methods	P (%)	R (%)	${\mathrm{mAP}}_{50}$ (%)	${\mathrm{mAP}}_{50:95}$ (%)	Param (M)	FLOPs (G)	Inference Time (ms)
RT-DETR [78]	91.0	89.4	93.0	58.7	41.9	125.6	12.1
YOLOv5n [68]	91.2	89.2	93.1	60.4	2.5	7.1	1.9
YOLOv8n [69]	**92.1**	88.6	93.3	60.9	3.0	8.1	1.9
YOLOv10n [70]	90.2	87.6	93.1	60.1	2.3	6.5	2.0
YOLOv11n [55]	91.1	89.3	93.3	61.0	2.6	6.5	**1.6**
YOLOv12n [79]	89.5	85.0	92.6	59.3	2.6	6.3	1.8
YOLOv13n [80]	90.7	88.1	93.4	59.8	2.5	6.1	1.9
YOLOv26n [71]	90.4	87.0	93.0	60.0	2.4	5.2	1.7
ITD-YOLOv8 [72]	—	—	93.5	—	1.8	6.0	—
G-YOLO [73]	—	—	91.4	—	0.8	3.7	—
LRI-YOLO [75]	90.7	89.1	94.1	—	1.6	3.8	—
ELNet [5]	91.5	90.1	**94.7**	60.5	**0.3**	**3.1**	—
Ours	91.7	**90.3**	94.5	**62.0**	0.4	4.6	**1.6**

Note: All inference time values are measured on an NVIDIA RTX 3090 GPU with an input resolution of 640×640 and a batch size of 16. Bold values denote the best performance in each metric. The em dash (—) indicates that the corresponding metric is not reported in the original literature.

**Table 10:** Performance comparison of various detectors on the VisDrone2019 dataset.

Methods	P (%)	R (%)	${\mathrm{mAP}}_{50}$ (%)	${\mathrm{mAP}}_{50:95}$ (%)	Param (M)	FLOPs (G)	Inference Time (ms)
RT-DETR [78]	**47.2**	**34.9**	**31.6**	**17.7**	41.9	125.7	21.3
YOLOv5n [68]	38.4	30.3	26.4	14.6	2.5	7.1	1.8
YOLOv8n [69]	38.2	29.6	26.8	14.9	3.0	8.1	1.9
YOLOv10n [70]	38.7	29.9	26.9	14.8	2.7	6.5	2.1
YOLOv11n [55]	38.6	29.4	27.2	15.1	2.6	6.5	**1.7**
YOLOv12n [79]	39.9	28.5	27.1	15.0	2.5	6.5	1.9
YOLOv13n [80]	38.8	28.3	26.9	14.9	2.5	6.3	1.9
YOLOv26n [71]	37.7	29.8	26.6	14.7	2.4	5.4	1.8
YOLOv5n+TDAM [74]	38.2	29.04	27.4	14.2	1.8	4.4	—
DLNet [76]	—	—	26.9	14.3	1.0	**1.6**	—
Drone-YOLO [77]	—	—	31	17.5	3.1	—	—
ELNet [5]	38.6	31.2	28.4	15.5	**0.3**	3.1	—
Ours	41.1	30.9	29.4	16.4	0.4	4.6	1.8

Note: All inference time values are measured on an NVIDIA RTX 3090 GPU with an input resolution of 640×640 and a batch size of 16. Bold values denote the best performance in each metric. The em dash (—) indicates that the corresponding metric is not reported in the original literature.

**Table 11:** Scale-wise detection results on VisDrone2019.

Methods	AP-Small (%)	AP-Medium (%)	AP-Large (%)
YOLOv11n [55]	5.8	22.4	33.4
YOLOv12n [79]	5.7	22.1	**33.6**
Ours	**7.4**	**24.4**	32.9

Note: Metrics follow the standard COCO protocol. AP-Small, AP-Medium, and AP-Large denote the precision for objects with small (<$32^{2}$ pixels), medium ($32^{2}$ to $96^{2}$ pixels), and large (≥$96^{2}$ pixels) scales. Bold values denote the best performance in each metric.

## Data Availability

The original contributions presented in this study are included in the article. Further inquiries can be directed to the corresponding author.
